# Extent of and trends in inequalities in child stunting in Sierra-Leone from 2005 to 2013: evidence from demographic and health surveys and multiple indicator cluster surveys

**DOI:** 10.1186/s12939-020-01212-5

**Published:** 2020-06-05

**Authors:** Gebretsadik Shibre, Betregiorigis Zegeye, Jemal Haidar

**Affiliations:** 1 Department of Reproductive, Family and Population Health, School of Public Health, Addis Ababa University, Addis Ababa, Ethiopia; 2 Birehan for mothers and children morbidity and mortality surveillance project, Debre Brehan, Ethiopia; 3 Department of Nutrition and Dietetics, School of Public Health, Addis Ababa University, Addis Ababa, Ethiopia

**Keywords:** Stunting, Inequality, Sierra Leone, DHS, MICS

## Abstract

**Background:**

Comprehensive assessment of stunting disparity in Sierra-Leone has not been done so far. We aimed to document extent and over time dynamics of inequality in stunting in Sierra-Leone using approaches that facilitate implementation of interventions aim to eliminate non-justified stunting disparity in the country.

**Methods:**

The data for the study were derived from two rounds of the Sierra Leone Demographic and Health Survey conducted in 2008 and 2013, and two rounds of the Sierra Leone Multiple Indicator Cluster Survey done in 2005 and 2010. We used the 2019 update WHO Health Equity Assessment Toolkit (HEAT) to do the analysis. The toolkit makes use of data stored in the WHO Health Equity Monitor database. We analyzed stunting inequality using summary measures: Population Attributable Risk, Population Attributable Fraction, Difference and Ratio. The summary measures were computed for five equity stratifers: wealth, education, child’s sex, place of residence and subnational region. We computed 95% Confidence Interval (CI) for each point estimate to show whether or not observed stunting inequalities are statistically significant, and whether or not the disparity changed over time statistically significantly.

**Results:**

The findings demonstrated stark inequalities in stunting in all the studied dimensions of inequality. While residence and subnational regional related inequalities remain unchanged over time, wealth and educational inequality had seen slight improvement during the same time period. Large sex related stunting inequality remained during the first three surveys time points, but it disappeared in 2013.

**Conclusions:**

Huge stunting disparities occurred in Sierra Leone, and the disparity disproportionately affects disadvantaged subpopulations and male children. Nutrition interventions that specifically target the subgroups which suffer more from the burden of stunting are required.

## Background

Stunting is a form of childhood undernutrition used to refer to population of children who are too short for their age and is an unambiguous sign of not developing well physically and mentally inside the first 1000 days [1]. Not only is stunting labeled as the “best overall indicator” of children’s well-being, but it is recognized as an “accurate reflection” of disparities among populations [2]. Stunting is also known to be “both a symptom of past deprivation and a predictor of future poverty” [3].

Child stunting is associated with numerous attributes such as poor socioeconomic status, insufficient nutritional intake, infectious diseases, and other environment factors [4, 5]. The lasting damage of stunting is not just confined to those who are stunted, but it also has serious consequences on the socioeconomic development of a nation [1]. Finally, stunting is associated with poor school performance and subsequently, leads to income inequality, high fertility, poor caring for children and to perpetuation of poverty across generations [6, 7]. Worldwide, 21.3% of under 5 children suffered from stunting in 2019 [8]. South Asia and Sub-Saharan Africa are the hardest hit places in the world, where one in three under five children is stunted [1]. In Africa, two in every five under five children are stunted, with large variations across the five sub-regions; it varied between 2% in Southern Africa to 23.1% in Eastern Africa [8]. Furthermore, not only is Africa home to a significant proportion of stunted children, but it is the only region that saw increased number of stunted children between 2000 and 2018 [1]. Like the situation in SSA, Sierra Leone experiences nutritional problems, and many people suffer from food insufficiency. Evidence shows that more than 3 million people in Sierra Leone are estimated to be deprived of access to food and among them, 170, 000 are estimated to be severely food insecure. Chronic malnutrition is a widespread problem in the country, with nearly one in three children suffers from stunting, and Sierra Leone is under the WHO “high” threshold category for stunting burden [9, 10].

It is worth noting at this junction that decline in national stunting prevalence is not necessarily associated with absence of within country stunting inequality across different subpopulation. Past studies observed the possibility for stunting disparity to still prevail in that country even when its overall prevalence is low [11]. This evidence points to the practical contributions of stunting inequality studies to the combat against its burden both nationally and worldwide. To this end, many studies have been conducted to determine whether within country variations exist in the prevalence of stunting between different subgroups. Children born to mothers who are economically worse-off, uneducated, residing in rural areas and in certain geographic locations are at elevated risk of stunting [12–16]. Moreover, it has been shown that male children are at a higher risk of stunting than their female counterparts [17, 18].

Measuring extent of disparities around a health care indicator is a decisive first step to locate where intervention is required, and design how health-related policies and strategies are implemented properly to help narrow the gap by addressing people who are left behind. Such endeavors could be achieved through analysis of data disaggregated by population subcategories into how numerous aspects of health are experienced in the entire population. The call for “data disaggregation” under the Sustainable Development Goal [19] has led to the proliferation of inequality studies on health care service globally. However, there is no in-depth evaluation of stunting inequality in Sierra Leone so far. We located few studies done on stunting in the country [20–25]. However, our study substantially updates the existing knowledge in many aspects. First, we looked at the over time dynamics of stunting disparity between 2005 and 2013 with respect to five different equity stratifiers. This would help policy makers and planners to learn the government’s past endeavors on stunting and to reframe future stunting interventions to end the disparity by focusing on those subpopulations suffering higher burden of stunting. Second, the analysis was performed using the high-quality WHO Health Equity Monitor (HEM) data. Finally, following the WHO recommendation for equity studies, we carried out the inequality analysis using simple and complex, as well as absolute and relative measures of inequality. This approach is important not to miss out disparities when they actually exist and also to capture disparities from different perspectives [26]. The study is specifically designed to document whether or not stunting inequality exist in Sierra Leone with respect to different dimensions of inequality with different inequality measures, and whether or not the disparity changed over time.

## Methods

### Study area

Sierra Leone has a total population of 7,092,113 with slightly more females than male population (3,601,135 females and 3,490,178 males, and nearly 6 in 10 people live in rural area [27]. The average annual population growth rate was 3.2% between 2004 and 2015 with a total fertility rate (TFR) of 5.2 children per woman. Sierra Leone has a young population with nearly 41% of the population being 15 years old or under [27]. Economy grew at 3.5% in 2018, and poverty is a widespread problem in the country, with more than half of the population living in the poverty zone. Until Ebola outbreak occurred in 2014, Sierra Leone was working to move into middle-income country by 2035, but problems of high youth unemployment, corruption and weak governance continue to pose greatest impediments to the country’s development [28]. The 2019 Global Hunger Index (GHI) report showed that Sierra Leone ranked 103rd with a score of 30.4, suggesting a serious levels of hunger in the country [29].

### Data

We used two rounds of the Sierra-Leone Demographic and Health Survey (SLDHS) data conducted in 2008, and 2013, and two rounds of the Sierra-Leone Multiple Indicator Cluster Survey (MICS) carried out in 2005 and 2010. The DHS and MICS are the major sources of information for monitoring key demographic and public health indicators and are highly comparable household surveys that permit direct comparison between them [30, 31]. They provide data on different background characteristics of respondents of a survey and health indicators including indicators of maternal, newborn, and child health in low-and-middle-income countries. The surveys cover different topics which include inter alia child mortality; maternal mortality; maternal health care services; child immunization coverage; childhood fever, acute respiratory infections, and diarrhea; HIV (knowledge of transmission and prevention, prior testing, stigma, and discrimination); and biomarkers (height and weight). Women age 15 to 49 and men age 15 to 59 were eligible for the surveys, and information was also gathered for under five children.

In 2005 and 2010 Sierra Leone MICS, 7654 and 13,416 women age 15 to 49 were interviewed respectively, and information was gathered from mothers or care givers of 5246 and 8619 under five children respectively. Similarly, in 2008 and 2013 SDHS, 7422 and 16,658 females age 15 to 49 respectively were interviewed. In addition, valid height and weight data were taken from 2770 and 5090 children under five in 2008 and 2013 SDHS respectively.

The SLDHS sample is designed to produce reliable estimates for important indicators for the entire country, as well as for urban and rural areas. Sample allocation and participant selection in the SLDHSs have been described in detail in the ‘appendix’ section of the SDHSs pdf final reports [22, 23]. Briefly, the samples were selected through stratified cluster sampling technique that happened in two stages. Each district in the country was first stratified in to urban and rural areas. Then samples were drawn independently from each stratum in two stages. In the first stage, enumeration areas (also called clusters or primary sampling units) were selected through probability proportional to size technique. Information about the enumeration areas was taken from the 2004 Sierra Leone Population and Housing Census (PHC) and the 2004 PHC provided sampling frame for the first stage. In the second stage, a fixed number of households were drawn from each enumeration area. Populations living in collective housing units such as hotels, hospitals, work camps, prisons, or boarding schools were excluded. The MICS follows a sampling strategy similar to that of DHS [24, 25].

### Variables

We measured inequality for child stunting among children who were born 5 years prior to the respective household surveys. Stunting is defined in standard deviation units (z-scores) from the median of the reference population. Children whose height-for-age-z score fall below minus 2 SD from the median of the WHO reference population are either moderately or severely stunted, and this definition is used in this study. We disaggregated stunting prevalence using five dimensions of inequality represented in the WHO Health Equity Monitor [32] which included wealth, education, place of residence, subnational region and child’s sex. Education is measured as three subgroups (no education, primary school and secondary school and higher). Wealth is classified into five categories: poorest, poor, middle, rich and richest. Wealth index has been used as a proxy for economic status in household surveys and is computed based on household assets and possessions using a statistical technique called Principal Component Analysis [33]. Residence is a binary variable and is measured as urban or rural and sex as male vs. female. Subnational region is measured as North, South, East and West.

### Statistical analysis

We assessed stunting inequalities following recommendation on health equity study of the WHO. Prevalence of stunting was first disaggregated by the five equity stratifiers discussed above. Then, the inequality was further assessed using four inequality measures: Difference (D), Population Attributable risk (PAR), Ratio (R) and Population Attributable Fraction (PAF).

Calculations for each summary measure have been detailed elsewhere [26, 34]. Briefly, PAR is calculated as the difference between the estimate for the reference subgroup *y*_*ref*_ and the national average μ: PAR = y_ref_ - μ. The selection of the reference subgroup *y*_*ref*_ depends on the type of health indicator and on the characteristics of the dimension of inequality, for which PAR is calculated. The health care indicator in this study is stunting and is unfavorable. Thus, for residence and sex dimensions, y_ref_ refers to the subgroups with the lowest estimate, which are respectively urban and female subcategories. For ordered dimensions (wealth and education in this study), y_ref_ refers to the most-advantaged subgroup (wealthiest and secondary education groups). For subnational region, y_ref_ refers to the subgroups with the lowest estimate which are different in the four rounds of the household surveys. PAF is calculated by dividing the population attributable risk (PAR) by the national average μ and multiplying the fraction by 100: PAF = [PAR / μ] * 100.

D is calculated as the difference between two subgroups: D = y_high_ - y_low_. For binary dimensions (residence and sex for our study), y_high_ refers to the subgroup with the highest estimate (rural and male) and y_low_ to the subgroup with the lowest estimate (urban and female). For ordered dimensions, y_high_ refers to the most-disadvantaged subgroup (poorest and illiterate subgroups) and y_low_ to the most-advantaged subgroup (richest and secondary education subgroups). For subnational region, y_high_ refers to the subgroup with the highest estimate and y_low_ to the subgroup with the lowest estimate. The calculation of R parallels that of D except that we divide estimate in one category by another category. See Table 1 in the result section to identify subgroups with highest and lowest estimates of stunting.

**Table 1:** Prevalence of childhood stunting across different dimensions of inequality in Sierra-Leone from 2005 to 2013

Dimensions of inequality	Year							
	2005		2008		2010		2013	
	%(95% CI)	Pop**n**	%(95% CI)	Pop**n**	%(95% CI)	Pop**n**	%(95% CI)	Pop**n**
**Wealth**								
Poorest	49.95 (46.40, 53.50)	1034	36.45 (31.88, 41.27)	604	46.84 (43.65, 50.06)	1725	42.63 (39.08, 46.26)	1182
Poor	52.53 (49.20, 55.84)	1150	43.59 (38.57, 48.76)	580	48.91 (45.73, 52.10)	1743	40.41 (36.52, 44.43)	1196
Middle	48.57 (45.15, 51.99)	1079	37.71 (32.54, 43.17)	615	47.84 (44.90, 50.78)	1611	38.14 (34.30, 42.14)	1121
Rich	45.53 (42.37, 48.72)	952	36.52 (30.91, 42.52)	589	41.46 (38.28, 44.72)	1519	35.02 (31.43, 38.78)	945
Richest	32.76 (29.03, 36.71)	697	22.65 (18.06, 28.01)	373	32.78 (29.13, 36.66)	1129	28.11 (23.49, 33.24)	647
**Education**								
No education	49.06 (47.19, 50.93)	3957	37.91 (34.96, 40.96)	1701	46.49 (44.55, 48.43)	5644	38.96 (36.60, 41.37)	2873
Primary school	40.08 (35.88, 44.43)	506	30.09 (23.74, 37.31)	234	40.33 (36.77, 43.99)	1024	38.39 (33.34, 43.70)	577
Secondary school +	35.80 (31.70, 40.10)	445	22.88 (17.53, 29.28)	212	37.27 (33.91, 40.75)	1061	32.54 (27.74, 37.73)	658
**Residence**								
Rural	49.21 (47.29, 51.14)	3874	38.87 (36.05, 41.76)	2013	45.74 (43.84, 47.64)	5620	40.31 (37.99, 42.66)	3923
Urban	38.54 (35.14, 42.05)	1040	29.70 (24.63, 35.32)	750	40.86 (37.05, 44.78)	2110	29.66 (26.47, 33.06)	1169
**Sex**								
Female	44.97 (42.77, 47.19)	2485	34.27 (31.44, 37.22)	1422	41.66 (39.56, 43.79)	3876	36.90 (34.42, 39.46)	2633
Male	48.99 (46.79, 51.19)	2429	38.62 (35.10, 42.26)	1341	47.17 (45.05, 49.29)	3854	38.89 (36.58, 41.24)	2460
**Regions**								
01 east	44.11 (40.38, 47.92)	1199	33.56 (29.09, 38.35)	507	41.50 (38.35, 44.73)	2067	42.17 (37.81, 46.66)	1182
02 north	52.39 (49.68, 55.09)	1887	39.51 (35.62, 43.54)	1327	48.64 (45.76, 51.53)	2930	35.40 (32.61, 38.30)	2226
03 south	45.79 (42.96, 48.64)	1390	38.05 (32.81, 43.57)	546	42.71 (39.99, 45.48)	1944	42.19 (38.26, 46.21)	1164
04 west	34.96 (30.65, 39.52)	436	26.89 (22.09, 32.29)	382	40.44 (34.24, 46.97)	787	28.91 (23.68, 34.76)	520

*Notes: popn Population share of each subgroup, CI Confidence Interval*

The PAR and PAF are complex measures of inequality that show potential for improvement in the national level of a health indicator if all sub-groups are performing equally with the best performing sub-group. They take positive values for favorable health intervention indicators and negative for adverse health indicators like stunting. The larger the absolute value of PAF and PAR, the larger the degree of inequality. They become zero if no further improvement can be achieved. On the other hand, D and R are simple measures of inequality that show gap between just two subgroups of an equity stratifier. If there is no inequality, D takes the value of zero and R becomes one. The further the value of D and R from zero and 1, respectively, the higher the level of inequality becomes.

While D and PAR are absolute measures, PAF and R are relative measures of inequality. Absolute measures show the absolute difference or gap in health or health care indicators between subgroups considered and retain the original units of the health indicators studied. Relative measures, on the other hand, measures disparities in relative terms.

Complex measures are able to account for sizes of all subgroups of an equity stratifer, not just two groups. This feature of complex measures of inequality makes them robust choice especially in the event of population shift. However, simple measures do not possess this property. While complex measures have the drawbacks of somehow challenging interpretation and calculation, simple measures have the strength of straightforward interpretation and calculation. WHO recommends adoption of simple and complex, as well as absolute and relative measures in an inequality study to allow for examination of an inequality from different perspectives [26].

We measured the over time dynamics of stunting disparity using the 95% Confidence Intervals (CI) approach. We declared that a statistically significant difference was observed when the different CIs do not overlap. On the other hand, when the intervals do overlap, we declared that there was no a statistically significant difference in inequality level between any two survey years. For all our analyses, we used the offline version of the WHO’s HEAT software application updated in 2019 [34]. The WHO HEM database stores re-analyzed data derived from DHS and MICS that have been conducted in many low-and-middle-income countries. The HEAT then uses the re-analyzed data in a way that facilitate interpretation of findings and is updated regularly to incorporate new surveys. Given that experts proficient in the area analyzed the data, papers written based on this source is deemed reliable and comparable both over time and between countries.

### Ethical consideration

Since the analyses were made using the publicly available data stored in the WHO HEM, we did not require ethical permissions. Ethical procedures were the responsibility of the institutions that commissioned, funded, or managed the surveys.

## Results

### Prevalence of childhood stunting disaggregated by different equity stratifiers

Table 1 presents the prevalence of stunting disaggregated by subcategories of the five dimensions of inequality for the four waves of the household surveys (two MICS-2005 and 2010, and two DHS-2008 and 2013). The stunting disaggregation is presented along with the population share of each subgroup in absolute numbers. The total children population participated in all the four rounds were 20, 500. Of these, 50.8, 75.2 and 69.1% were females, rural residents and born to illiterate women, respectively. We presented stunting point estimate in each subgroup along with the corresponding 95% CIs to allow comparison between subgroups and over time. For example, the prevalence of stunting varied significantly between wealth categories (the 95% CIs of the five wealth categories did not overlap), with the richest category contributed the smallest portion of stunting burden. While stunting among the poorest subgroup had fallen by about 7 percentage points between 2005 and 2013, the CIs for the richest subgroup had substantial overlap, making it difficult to comment on the rate of reduction of stunting. Similarly, while stunting burden in the no-education group had decreased by 10 percentage points over the study time periods, the other two categories of education had seen no statistically significant change over time. We found that prevalence of stunting was consistently higher among male children and rural residents throughout the studied years. See Table 1 for detail.

### Inequalities in stunting as measured by different inequality measures

As shown in Table 2, there were both absolute and relative wealth related inequalities in stunting in Sierra-Leone in all the four rounds of the surveys and with all the measures of disparity, with the inequality occurred consistently favouring economically better-off groups. For example, in 2005, based on just a point estimate, the prevalence of stunting among children born to poorest women was nearly 17 points higher than the prevalence in children born to women who fell in the richest wealth quintile The R showed that children from poorest households had stunting that is between 1.3 to 1.7 times higher than that of children from richest households in 2005.

**Table 2:** Extent and trends of inequalities in childhood stunting across different measures of inequalities from 2005 to 2013

Dimensions of inequality	Year				
		2005	2008	2010	2013
	Measure	%(95% CI)	%(95% CI)	%(95% CI)	%(95% CI)
Wealth	D	17.19 (11.97, 22.41)	13.79 (6.96, 20.61)	14.05 (9.11, 18.99)	14.52 (8.47, 20.56)
	PAF	−30.23 (− 37.20, −23.26)	−37.72 (−48.86, −26.59)	−26.16 (− 31.93, − 20.39)	−25.76(− 34.41, −17.10)
	PAR	−14.19 (− 17.47, − 10.92)	−13.72 (− 17.77, − 9.67)	− 11.61 (− 14.18, − 9.05)	−9.75 (− 13.03, −6.47)
	R	1.52 (1.31, 1.73)	1.60 (1.20, 2.01	1.42 (1.23, 1.61)	1.51 (1.22, 1.80)
Education	D	13.26 (8.67, 17.85)	15.03 (8.45, 21.60)	9.21 (5.28, 13.14)	6.42 (0.89, 11.95)
	PAF	−23.72 (− 32.83, −14.61)	− 35.67 (− 51.07, − 20.26)	− 16.07 (− 22.19, − 9.94)	− 14.03 (− 22.77, − 5.30)
	PAR	− 11.13 (− 15.40, − 6.85)	−12.68 (− 18.16, − 7.20)	−7.13 (− 9.85, − 4.41)	− 5.31 (− 8.62, − 2.00)
	R	1.37 (1.20, 1.53)	1.65 (1.21, 2.10)	1.24 (1.12, 1.37)	1.19 (0.99, 1.39)
Residence	D	10.67 (6.72, 14.62)	9.16 (3.11, 15.22)	4.87 (0.57, 9.17)	10.64 (6.61, 14.67)
	PAF	−17.92 (− 23.56, − 12.27)	−18.35 (− 26.18, − 10.52)	−7.98 (− 12.03, − 3.93)	−21.66(− 27.84, − 15.47)
	PAR	−8.41 (− 11.06, − 5.76)	−6.67 (− 9.52, − 3.82)	−3.54 (− 5.34, − 1.74)	− 8.20 (− 10.54, − 5.85)
	R	1.27 (1.15, 1.40)	1.30 (1.05, 1.56)	1.11 (1.00, 1.23)	1.35 (1.18, 1.52)
Sex	D	4.01 (0.90, 7.12)	4.34 (−0.23, 8.93)	5.50 (2.51, 8.49)	1.98 (−1.44, 5.41)
	PAF	− 4.22 (− 7.17, − 1.28)	− 5.80 (− 10.59, −1.00)	− 6.18 (− 8.67, − 3.69)	−2.52 (− 5.93, 0.87)
	PAR	−1.98 (− 3.36, − 0.60)	−2.11 (− 3.85, − 0.36)	−2.74 (− 3.85, −1.63)	−0.95 (− 2.24, 0.33)
	R	1.08 (1.01, 1.16)	1.12 (0.98, 1.26)	1.13 (1.05, 1.20)	1.05 (0.95, 1.14)
Regions	D	17.43 (12.25, 22.61)	12.62 (6.17, 19.06)	8.19 (1.19, 15.20)	13.27 (6.46, 20.09)
	PAF	−25.55 (− 34.71, − 16.38)	− 26.08 (− 37.61, − 14.56)	−8.92 (− 16.25, −1.58)	− 23.64 (− 33.49, − 13.79)
	PAR	−11.99 (−16.30, − 7.69)	− 9.49 (− 13.68, − 5.29)	−3.96 (− 7.21, − 0.70)	− 8.95 (− 12.68, − 5.22)
	R	1.49 (1.29, 1.70)	1.46 (1.15, 1.78)	1.20 (0.99, 1.40)	1.45 (1.14, 1.77)

Notes: *D* Difference, *PAF* Population Atrributable Fraction, *PAR* Population Attributable Risk, *R* Ratio, *CI* Confidence Interval

The pro-rich condition of stunting has also been supported by the complex measures. For example, the PAF finding showed that stunting was more concentrated among the poor in all the four survey periods, and that prevalence of stunting in the country in 2008 would have been fallen between roughly 27 to 49% if the poorest, poor, middle and rich subgroups of wealth were on par with the richest subgroup with respect to stunting burden. Although the disparity do not changed noticeably by the simple measures, the complex measures indicated that the wealth related disparity in stunting improved slightly over time though overlap of the CIs complicate interpretation of the change.

Likewise, educational status inequality in childhood stunting was observed across all the four rounds. We found education related disparity in 2005, 2008 and 2010 schools by all the summary measures and in 2013 by PAR, PAF and D, favouring children born to mothers with secondary or higher schools. Based on the point estimates, stunting among children born to non-educated women was roughly 13 points higher than stunting in children born to women who completed secondary education or higher in 2005. This difference was halved in 2013. Over the surveyed years, there had been small decline in the educational disparity of stunting. For example, over the period of 8 years, stunting decreased by nearly 10 points according to the point estimate of the PAF measure.

Our findings also demonstrated that, except in 2010 by the R measure, where there was no disparity, there was place of residence inequality in stunting in all the rounds and the disparity disproportionately affected the rural children. The point estimates from D showed that children in rural areas had nearly 11, 9, 5 and 11 percentage points higher stunting burden than that of their counterparts in urban areas in 2005, 2008, 2010 and 2013 respectively, yielding more like a ‘U’ shape pattern. The urban-rural stunting disparity had seen fluctuations; it decreased between 2005 and 2010 by 10 points, and increased afterwards by 14 points as evidenced by the PAF.

While we did not record any male-female differential in the occurrence of stunting in 2013, we highlighted large sex related inequality in stunting in the first three household surveys, where we observed the disparity in 2005 and 2010 by all the measures and in 2008 by the complex measures only. If there was no male-female gap in stunting (or equivalently, if stunting level in male children was reduced to a level in females), then stunting in the country would have been decreased by between nearly 1 to 7 points in 2005, nearly 1 to 11 points in 2008, and nearly 4 to 9 points in 2010.

In terms of subnational region, except in 2010 by simple measure (R), where there was disparity, there were both absolute and relative subnational regional inequalities in childhood stunting across all the four rounds. The regional variations of stunting had decreased between 2005 and 2010 by D, PAF and PAR measures, and increased between 2010 and 2013 by the complex measures.

## Discussion

The study attempted to shed light on the over time dynamics of inequalities in the prevalence of stunting in Sierra-Leone among children aged below 5 years using the WHO HEM database.

Our findings demonstrated that both absolute and relative economic inequalities in childhood stunting were prevalent in all the four household surveys. The stunting burden was more pronounced among the most disadvantaged sub-groups who fell under the poorest wealth categories. The poor-rich disparity in stunting could be due to dissimilarity in the distribution of income, education, and ethnicity that are directly or indirectly related with food security [24]. The fact that the pro-rich situation of stunting inequality did not improve significantly over time raises a concern on whether the country could achieve the stunting targets set for 2025 and 2030 SDG [35, 36]. The present findings are comparable with that of previous studies done in other African countries which reported that childhood stunting was greater among families who had low family income [37–39].

With regard to time trend of wealth driven stunting disparity, we confirmed that the pro-rich inequality of stunting had seen mixed patterns based on the type of summary measures calculated; while simple measures showed no sign of improvement, complex measures demonstrated that the wealth related inequality of stunting had slightly narrowed with time. As is clearly detailed in the method section of this paper, simple measures of inequality account for just two subgroups of a dimension of inequality, and ignore the remaining subpopulations in the middle. On the other hand, complex measures take into account the entire subgroups of an equity stratifer and this statistical property allows them to overcome the inherent blemish of simple measures when one wants to observe distribution of a health indicator along the entire rungs of a dimension of inequality like wealth and education. This difference between them could explain for the emergence of differing conclusion on the over time change of stunting disparity. In our study, the PAR showed that the wealth related stunting inequality decreased, based on the point estimate, by 4.4 percentage points between the first and the last surveys, without a statistically significant difference between the first three rounds. The PAR finding further showed that Sierra Leone would have reduced the national stunting prevalence by between, based on the point estimates, nearly 10 to 14 points had the levels of stunting in the other wealth quintiles been reduced to the level in the wealthiest quintile. Subsequently, the significant drop in the national prevalence of stunting could have been accompanied by large number of children escaped from the suffering of stunting. This in turn would have big effect on attainment of the national and global goals of stunting set for 2025 and 2030. Currently, one in 3 children in the nation lives with stunting [9], making Sierra Leone one of the countries with the hardest hit of stunting and it is unlikely for the country to hit the global targets for stunting unless pro-poor interventions are designed and promoted. This is because reducing income inequality and increasing health care spending have been shown to reduce stunting level [40]. Similarly, we documented that the relative disparity (according to the PAF finding) narrowed between the first and the last surveys studied at least based on the point estimates. Similar wealth related disparity in stunting was documented in literature [41]. A multi-country analysis based on DHSs conducted in 35 SSA countries showed that stunting was more concentrated among the poorer households [42]. Akombi BJ et al. (2019) indicated that children born to women who fell in the richest wealth category had, on average, a 47% lower odd of experiencing stunting compared with children born to women in the poorest wealth quintile [39]. Although exploring drivers of the change in stunting inequality over time is beyond the remit of the study, our finding argues that the differential pace of reduction of stunting over time between the poorest and richest may explain the fact that the poor-rich disparity narrowed in 2013. While the poorest subgroup recorded a 7 percentage point decline in the prevalence of stunting, the richest subgroup did not see any change in the prevalence of stunting during the same time (Table 1).

As all the summary measures calculated indicated, the burden of stunting in our study was highly concentrated among children born to women with no formal education than children born to women who had completed primary and secondary educations. Unfortunately, the education driven disparity persisted though evidence of improvement was observed. The simple measures indicated a statistically significant reduction of the education driven disparity over time including total disappearance in 2013 (by the measure of R). The complex measures also supported the finding that stunting disparity between educated and non-educated groups narrowed over time. We observed that over the period of the study, on average, the gap narrowed by 6 points and this is slightly higher than recorded in the wealth-based inequality. The absolute inequality based on the PAR finding showed that, it would have been possible to drop the country’s 2013 stunting prevalence by approximately 2 to 9 points if the prevalence of stunting among the non-educated subgroup had been reduced to the level in the secondary or above category. In terms of relative inequality finding derived from PAF, Sierra Leone was to reduce the national stunting level in 2013 by between closely 5 to 23% if there were no education related disparity in stunting. The finding points to the demonstrated practical implication of education on ensuring health care equity by breaking the vicious cycle of poverty [43]. In the effort to curb the threat of stunting by removing within country disparities, closing gap in secondary or more education is important. In this regard, Sierra Leone would be benefited from accelerated education program as the net enrollment rate is only 38.33% for secondary education [44].

Our finding that stunting is more common among children of women with low educational attainment is aligned with similar prior findings [16, 39, 42]. The possible explanation for this is that a higher maternal education could lead to improved health care use which, in turn, affects health-related decisions that improve child nutritional outcomes [45]. Like wealth related inequality, educational disparity in stunting had shown slight improvement over the course of the studied years. The same reason that we used to justify the improvement of wealth based disparity can be used to justify the improvement in educational inequality; variation in the rate of reduction of stunting between these subpopulations causes for the disparity to shrink; stunting decreased significantly over time in the non-educated category while it remained relatively unchanged among higher or more educated group (Table 1).

We showed that more stunted children were from rural than urban settings in all the four rounds and this evidence is supported by all measures. Our finding is similar with past evidence [39]. Such variations are likely to be due to the differences in the distribution of socioeconomic conditions [46]. The study highlighted not only was stunting highly widespread in rural areas, but the country had not seen any sign of narrowing in the urban-rural disparity over the studied years. We observed a roughly U shape pattern in the residence related inequality of stunting; by all but one measures, the disparity was lowest in 2010 with the first and the last survey years experiencing the highest disparity. More interestingly, the disparity disappeared in 2010 altogether by the R measure. It is not coincidence that all the measures identified 2010 as most equitable year than others, and further studies may be required to explore the reason. If rural areas were on par with urban settings, then there were substantial improvements in burden of stunting in the country. In 2013, for instance, Sierra Leone would have decreased the national stunting level by about 6 to 11 percentage points. In terms of the relative gains, the 2013 national stunting prevalence would have been reduced by nearly 15 to 28%. This finding emphasizes the need to draw increased attention towards rural areas if the aim is to close the urban-rural gap in terms of stunting burden. Policy makers and programmers should prioritize pro-rural and pro-poor nutritional interventions. Another important finding that emerges from our study is that variations in the level of stunting were observed among sub-national regions by all measures of inequality across all studied years, except that the disparity vanished in 2010 by the R measure. Generally, the regional inequality had shown fluctuation over time by the different measures of inequality; it fluctuated with complex measures; it decreased until 2010 and increased again afterwards, resulting in a U shape nature of the disparity. This is similar with pattern of wealth based disparity elucidated above. The fluctuation with the simple measure (D) was that it first decreased between 2005 and 2010, and remained unchanged afterwards.

What is particularly impressing is that the Northern and West regions host respectively the highest and lowest prevalence of stunting in the nation, causing stark regional variations in stunting to exist. It is important to explore why the Northern Sierra Leone contributed to the unacceptably high clustering of stunted children than any other parts of the country; identifying the drivers of this could pave the way to implementing context specific interventions that would help eliminate the subnational region related stunting disparity. Also, experiences that worked in the West of the country could be applied to the other settings in order to narrow the gap between regions and subsequently decrease the national stunting prevalence. The absolute measure indicated that Sierra Leone would have declined the 2005 stunting level in country between nearly 8 to 16 points if the prevalence of stunting in the North had reduced to the level in the west. Further, the nation was to reduce the 2013 stunting level between nearly 5 to 13 points if stunting prevalence in the South fell to the level in the West. The finding points to the need to launch specific policy relevant equity interventions that work best in the context of each region in the country if the aim is to accelerate attainment of global goals for stunting set for 2025 and 2030 [35, 36]. The present finding is in concordance with that of prior study which reported within country variations of stunting with respect to subnational regions [39].

Congruent with prior works [39, 47], our finding demonstrated glaring sex related stunting inequality favouring female children. A study revealed that female children had 16 to 25% lower odd of experiencing stunting than their male counterparts [39], supporting the pro-female nature of stunting observed in this study. While a decomposition analysis would be warranted to learn factors that contributed to the observed male-female gap with respect to stunting distribution, available evidence showed that mothers may be motivated to start Complementary Feeding (CF) early for boys when they are born small or “weak” due to pre-existing nutritional status, and weaker babies are likely to be breast fed for longer period without having CF when it should be initiated at a recommended age [37, 48]. This, in turn, results in weaker babies experiencing higher odd of stunting. However, the sex differential of stunting ended in 2013, where stunting equally affected both sexes. Even if the disappearance of pro-female nature of stunting in the recent survey might reflect the country’s commitment to end male-female disparity of nutritional problems, this issue warrants further investigation and the strategies that work for sex driven disparity can be applied to the socio-economic and residence related disparities of stunting to avoid within country variations in stunting burden.

The WHO nutrition targets call for 40 and 50% reduction of stunting in 2025 and 2030 respectively from the 2012 stunting level. On average, stunting has to be reduced by 4% annually to attain the stated targets [35, 36]. However, Sierra Leone is far removed from the stated average annual rate of reduction, with stunting decreased by a meager of only 0.25% annually between 2005 and 2013 [23, 24]. This means that the country needs to increase the observed stunting average annual rate of reduction by nearly 8 fold if the aim is to achieve the 14.7% stunting level by 2025 [36]. We argue that removing the large within country disparities around stunting between different subpopulations could significantly reduce the overall stunting prevalence, which in turn would translate to achievement of the global goals of stunting.

The study has a few strengths. We investigated the stunting disparity using the WHO HEM database through the HEAT software which allows us to do the inequality analysis with high standard of quality. The reason being, the re-a-analysis of the DHS and MICS data in the HEM was carried out by experts in the sector of the health equity and with huge care. Findings analyzed based on the HEM database are easily comparable across countries and over time. Finally the use of different summary measures in a single study has the potential to capture inequality from different ethical positions and perspectives, and this in turn affects the type of policy decisions and programmes to be made. The study also suffers a limitation. We did not explore the reason why stunting disparity remained in the country. Once it is known that stunting disparity exists in the country across different population groups, further researches are important to help explore factors that led to the inequality.

## Conclusions

Stark inequalities in stunting in Sierra-Leone remained to the disadvantage of children of poor and uneducated mothers, those living rural residents and certain regions. We also wed that stunting disproportionately impacted male children. Not only did we show the occurrence of substantial inequality to the disadvantage of certain subpopulations, we highlighted that the inequality persisted throughout the study periods though some signs of improvements were observed for some dimensions and the male-female stunting gap disappeared in 2013.

We recommend implementation of equitable nutrition interventions focusing on the sub-populations suffering higher burden of stunting. The country needs to increase the distribution and coverage of secondary or higher educations to close gaps in stunting and other SDG indicators between educated and non-educated individuals. Also, the glaring wealth disparity in the country should be the government’s top priority area to make economic schemes pro-poor. Finally, we recommend that the nation should work aggressively to ensure that people living in the poverty zone are benefiting from the country’s economic growth to ensure that they are not left behind.

## Data Availability

The datasets generated and/or analyzed during the current study are available in the WHO’s HEAT version 3.1 [https://www.who.int/gho/health_equity/assessment_toolkit/en/].

## Abbreviations

CI: Confidence Interval
D: Difference
DHS: Demographic and Health Survey
EA: Enumeration Area
GHI: Global Hunger Index
HEAT: Health Equity Assessment Toolkit
ICF: Inner City Fund
MICS: Multiple Indicator Cluster Survey
PAR: Population Attributable Fraction
PAR: Population Attributable Risk
PCA: Principal Component Analysis
PSUs: Primary Sampling Units
PPS: Probability Proportional to Size
R: Ratio
SDG: Sustainable Development Goal
SDHS: Sierra Leone Demographic and Health Survey
WHO: World Health Organization
